# Mitochondrial protection and anti-inflammatory effect of curcumin in inhibiting reproductive toxicity induced by sodium valproate in male mice

**DOI:** 10.22038/ijbms.2025.82254.17791

**Published:** 2025

**Authors:** Moein Shaneh, Milad Chahardori, Fereshte Talebpour Amiri, Nahid Amani, Fatemeh Shaki

**Affiliations:** 1 Department of Toxicology and Pharmacology, Faculty of Pharmacy, Mazandaran University of Medical Sciences, Sari, Iran; 2 Student Research Committee, Mazandaran University of Medical Sciences, Sari, Iran; 3 Department of Anatomy, Faculty of Medicine, Molecular and Cell Biology Research Center, Mazandaran University of Medical Sciences, Sari, Iran

**Keywords:** Curcumin, Inflammation, Mitochondria, Oxidative stress, Sodium valproate, Testis toxicity

## Abstract

**Objective(s):**

Sodium valproate (VPA) has harmful effects on the male reproductive system. The present study aimed to investigate the influence of curcumin (CUR) in mitigating the VPA-induced reproductive toxicity in male mice.

**Materials and Methods:**

The male mice (mean weight 20 g and 8 weeks old) were divided into six groups (n=6): control, VPA only (500 mg/kg, IP), VPA plus different doses of CUR (25, 50, and 100 mg/kg, IP), CUR alone (100 mg/kg, IP). After treatment for eight consecutive weeks, the mice were sacrificed, testicle tissues were separated, and mitochondria were isolated with different centrifuge techniques. Various biomarkers were evaluated in testis tissue, including the concentration of lipid peroxidation, glutathione, protein carbonyl, nitric oxide, IL-6, and TNF-alpha. Also, mitochondrial toxicity, swelling, and membrane potential were assessed. Furthermore, sperm analysis and histopathological examination were done on testicular tissue.

**Results:**

VPA injection increased the amount of nitric oxide, inflammatory factors, mitochondrial toxicity, and oxidative stress markers (*P<*0.05). Also, histopathological and sperm analysis showed significant damage to testis tissue and a significant reduction in sperm count, motility, and normal morphology after VPA administration. CUR led to a substantial reduction of the inflammatory and oxidative stress parameters(*P<*0.05), restored the VPA-induced testis toxicity, and increased sperm count and motility (*P<*0.05).

**Conclusion:**

Our study demonstrates CUR’s ameliorative effects on mitochondrial oxidative damage and inflammation caused by VPA-induced reproductive toxicity, which can be suggested as a strategy for reducing the side effects caused by VPA.

## Introduction

Valproic acid (VPA) is a derivative of naturally occurring valeric acid and belongs to the branched short-chain fatty acids class (1). VPA is primarily prescribed for treating epilepsy, seizure disorders (2), manic episodes, prevention of migraines, schizophrenia, major depression, and infantile spasms. (3-5) VPA exerts its therapeutic effects through multiple mechanisms of action, including blockage of voltage-dependent sodium channels, amplification of the gamma-aminobutyric acid (GABA)-ergic system, and elevating GABA levels(6-8). VPA is an inhibitor of voltage-gated calcium channels, particularly T-type channels, crucial in neuronal excitability and linked to thalamic bursts in absence seizures. By blocking calcium ion influx during neuronal depolarization, VPA reduces excessive neuronal firing and disrupts the necessary synchronization for seizure propagation. This modulation effectively suppresses seizure activity, especially in the absence of seizures characterized by T-type channel dysfunction (9). VPA affects potassium channels, though this is a lesser mechanism of the drug. It blocks specific potassium channels, reducing potassium efflux during repolarization, which prolongs neuronal depolarization and diminishes hyperpolarization. While the exact subtypes affected are not fully defined, they may include delayed rectifier potassium channels. By blocking these channels, VPA stabilizes the resting membrane potential and inhibits excessive neuronal firing, contributing to its antiepileptic properties (10).

Prolonged administration of VPA is associated with hepatotoxicity and gastrointestinal adverse effects like nausea, vomiting, and diarrhea. Additionally, it can cause neurological manifestations such as tremors, dizziness, and sedation. Reproductive complications linked to VPA include menstrual irregularities, polycystic ovary syndrome, and reduced fertility in males (11-13). According to reports, VPA therapy can cause infertility in male animals by affecting sperm count and motility (14, 15). Furthermore, studies conducted on animals have revealed that prolonged use of VPA can impact fertility and result in abnormal sperm morphology, leading to a decrease in both sperm quality and testosterone levels (16, 17).

According to previous research findings, it has been proposed that the induction of inflammation and oxidative stress are two significant pathways underlying the toxicity associated with VPA (18, 19). Research has demonstrated that VPA has the potential to alter the composition of the mitochondrial membrane, which can reduce mitochondrial functionality and, subsequently, mitochondrial respiration (20-22). Mitochondria are the primary sites of ATP synthesis and also produce reactive oxygen species (ROS)(23, 24). Consequently, any disruption to mitochondrial function can result in heightened ROS generation, oxidation of glutathione, peroxidation of lipids, and, ultimately, damage to cellular membranes (25).

The plasma membranes of spermatozoa are highly vulnerable to damage caused by ROS. When ROS levels exceed normal limits, it can lead to lipid peroxidation, which disrupts the integrity of the membrane and impairs sperm motility (26). Additionally, the proper functioning of sperm relies on the energy provided by the mitochondria (27). Hence, any disturbances in mitochondrial activity are strongly associated with disorders in sperm motility and overall sperm function (28).

The connection between oxidative stress and inflammation is reciprocal and interrelated. Compelling evidence supports that ROS can promote inflammation in various tissues by producing inflammatory mediators. In turn, inflammatory processes can produce ROS, which can cause cellular damage, activate redox-sensitive transcription factors, promote the release of pro-inflammatory cytokines, and impair antioxidant defenses. One specific molecule involved in inflammation is Tumor Necrosis Factor-alpha (TNF-α). TNF-α initiates the synthesis of additional pro-inflammatory substances, attracts immune cells toward the inflammation site, and facilitates tissue harm (29, 30).

Curcumin (CUR) is an organic compound extracted from the root of *Curcuma longa L.,* commonly known as turmeric. This natural compound possesses antioxidative and anti-inflammatory characteristics (22) and has long been utilized in Asian nations as a medicinal herb, owing to its multiple health-enhancing properties. CUR exhibits various advantageous medicinal attributes, including anti-inflammatory, anti-cancer, antifungal, anti-ischemic, anti-tumor, analgesic, anti-arthritic, and anti-mutagenic effects (24, 25). Earlier studies have provided evidence suggesting that CUR can protect laboratory animals against testicular damage induced by diverse toxins, such as cadmium and metronidazole (31, 32). 

CUR was found to have anti-apoptotic effects on spermatogenic cells and stimulate the growth and expansion of testicular tissue. Studies have also shown that CUR can alleviate the adverse effects of substances like Dianabol, promoting testicular volume and weight recovery (33)

In this study, we investigated the impact of mitochondrial oxidative damage and inflammation on VPA-induced reproductive toxicity. Additionally, we examined the potential effects of CUR in mitigating VPA-induced reproductive toxicity by evaluating its anti-inflammatory and mitochondrial protective properties.

## Materials and Methods

### Chemicals and reagents

All chemicals utilized in this study were of the highest quality and were purchased from Merck Germany and Sigma-Aldrich America. The solvents employed were HPLC grade, indicating the highest possible pharmaceutical grade obtainable. VPA and CUR were obtained from Sigma Aldrich (St. Louis, MO, USA).

### Animal treatment and study design

Male Mice (8 weeks old; 20±2 g ) were obtained from the Laboratory Animals Research Center, Mazandaran University of Medical Sciences, Sari, Iran. They were kept under standardized conditions of 24±1 ^°^C temperature and 55±5% relative humidity. They were given unrestricted access to food and water and exposed to a 12-hour light and 12-hour dark cycle. All animals were acclimatized for 7 days to the laboratory conditions before experimentation. These conditions were maintained throughout the study to ensure consistency, and no deviations were observed.

The experimental procedures were approved by the Committee for Animal Experimentation of Mazandaran University of Medical Sciences, Sari, Iran, under the ethical code IR.MAZUMS.REC.1398.826. These procedures align with the standards outlined in the US National Institutes of Health Guide for the Care and Use of Laboratory Animals (34).

A total of six groups of mice were used, each consisting of six animals. The first group serving as the control received saline and was given the solvent to dissolve VPA and curcumin (0.1% Tween-80). The remaining groups were treated with VPA and varying doses of curcumin, either alone or in combination. The interventions were given intraperitoneally once every day for six consecutive weeks. The groups were treated according to the following schedule: Group 1 (control) received normal saline, Group 2 received VPA alone [500 mg/kg], Group 3 received VPA [500 mg/kg]+curcumin [25 mg/kg (IP)], Group 4 received VPA[500 mg/kg]+curcumin [50 mg/kg (IP)], Group 5 received VPA [500 mg/kg]+curcumin [100 mg/kg (IP)], Group 6 received curcumin alone [100 mg/kg (IP)] with the vehicle for 8 weeks.

### Sample collection and preparation

The animals were sacrificed 24 hr after the final injection. The animals were anesthetized with ketamine at a dose of 87 mg/kg and xylazine at a dose of 13 mg/kg intraperitoneally under sterile conditions. Blood samples were promptly collected from the animals’ hearts and centrifuged at 3500×g for ten minutes at 4 ^°^C. The supernatants obtained were stored at -20 ^°^C for further analysis. Subsequently, the testis tissues were extracted and homogenized using phosphate-buffered saline at a volume equivalent to five times the amount of tissue. Following homogenization, the resulting mixture was centrifuged at 1000×g for ten minutes at a temperature of 4 ^°^C. This step allowed for the separation of nuclear and cellular debris, with the resulting supernatant being stored at -20 ^°^C for subsequent assessment of oxidative stress markers (35). 

### Biochemical assays in tissue

*Total protein content*

The Bradford method was utilized to quantify the protein content in the testis.

*Measurement of lipid peroxidation (LPO)*

The Zhang method (2008) was used to measure the concentration of malondialdehyde (MDA). To perform the measurement, 0.2 ml of the testis tissue homogenate was mixed with 0.25 ml of 0.05 M phosphoric acid and 0.3 ml of 0.2% thiobarbituric acid (TBA). All samples were incubated in a hot-water bath for 30 min and subsequently transferred to an ice bath. Next, 0.4 ml of n-butanol was added to each tube. The tubes were then centrifuged at 3500 rpm for ten minutes. Finally, the MDA level in each sample was determined by measuring the absorbance of the resulting supernatant at a wavelength of 532 nm using an ELISA reader (Tecan, Rainbow Thermo, Austria). Tetramethoxypropane (TEP) served as the reference substance, with the findings presented in micromolar (μM)/mg of protein (36).

*Measurement of glutathione content*

Glutathione (GSH) was quantified using 5,5’-dithio-bis-[2-nitrobenzoic acid] (DTNB) as an index. In brief, 0.1 ml of testis tissue was mixed with 0.1 mol/l of phosphate buffers, and 0.04% DTNB was prepared to obtain a total volume of 3.0 ml with a pH of 7.4. The yellow color produced was analyzed using a spectrophotometer (UV-1601 PC, Shimadzu, Japan) at 412 nm. The concentration of GSH was measured in nM (37).

*Protein Carbonyl Concentration*

The 2,4-dinitrophenylhydrazine (DNPH) assay was implemented to quantify the protein carbonyl. In summary, 200 µl of samples were mixed with 500 µl of 20% w/v TCA and stored at 4 ^°^C for 15 min. Next, 500 µl of 0.2% DNPH was added to the samples, while 2 M HCl was added to the control group. After centrifugation, the microtubes underwent a rinsing process using a 1000 µl solution composed of ethanol and ethyl acetate in a 1:1 volume-to-volume ratio. Afterward, the samples were dissolved in 200 µl of 6 M guanidine hydrochloride, and the absorbance at 365 nm was measured to quantify the carbonyl content. (38)

*Measurement of nitric oxide*

Nitric oxide (NO) levels were assessed using commercially available Griess reagent kits. This method involves the conversion of sulfanilic acid to a diazonium salt through a reaction with nitrite in an acidic solution. The diazonium salt is then coupled with N-(1-naphthyl) ethylenediamine to produce an azo dye. The absorption of this dye at 548 nm is measured using a spectrophotometer (39).

*Measurement of TNF-alpha and Interleukin 6 (IL-6)*

TNF-α and IL-6 serum levels were measured using an enzyme-linked immunosorbent assay kit (ELISA). In brief, the process involved the addition of standard, control, and mouse serum into wells previously coated with a monoclonal antibody designed to target mouse TNF-α or IL6 following the binding of TNF-α or IL6 to the immobilized antibody; a washing step eliminated any unbound remaining compounds. Each well was rinsed to remove any unbound antibody-enzyme reagent after adding an enzyme-linked polyclonal antibody specific for mouse TNF-α or IL6. Ultimately, each well was filled with a substrate solution, which caused an enzymatic reaction that produced a blue product. Upon adding the stop dilution, the blue product turned yellow (40).

### Mitochondrial function assay

*Isolation of mitochondria*

Differential centrifugation was required for the separation of mitochondria from homogenized tissues. Initially, the homogenates were centrifugated at 1000 ×g for eight minutes at a temperature of 4 ^°^C. The resulting supernatants were carefully gathered and then subjected to a second round of centrifugation at 10,000 ×g for ten minutes at 4 ^°^C. The obtained pellets were then resuspended in an isolation buffer and underwent a further centrifugation step at 12,300 ×g for ten minutes at 4 ^°^C. After the supernatants were collected, they were transferred and supplemented with isolation buffer containing ethylene glycol tetraacetic acid (EGTA), which consists of 215 mM mannitol, 75 mM sucrose, 0.1% Bovine serum albumin (BSA), 20 mM Hydroxyethylpiperazine Ethane Sulfonic Acid (HEPES), 1.0 μM EGTA, and adjusted to a pH of 7.4 with KOH. Afterward, the mixture was centrifuged at 12,300g at 4 ^°^C for ten minutes. The pellets obtained at this stage contained the purified mitochondria and were resuspended in the isolation buffer. All the procedures were conducted under ice-cooled conditions to maintain a constant low temperature throughout the protocol (41).

*Determination of mitochondrial function*

Mitochondrial function was assessed by 3-(4,5-dimethylthiazol-2-yl)-2,5-diphenyltetrazolium bromide (MTT) test. In mitochondria, succinate dehydrogenase reduces this yellow indicator to purple formazan. After mitochondrial Isolation, MTT (0.4%) was added to mitochondria and incubated at 25 ^°^C for 30 min. The product of purple formazan crystals was dissolved in 100 μl DMSO, and the absorbance at 570 nm was quantified using an ELISA reader (Tecan, Rainbow Thermo, Austria) (42).

*Measurement of mitochondrial membrane potential*

To determine the mitochondrial membrane potential (MMP), rhodamine 123(Rh123), a cationic fluorescent dye, was employed. The mitochondrial fractions containing 1 mg protein/ml were incubated with 10 µM of Rh123 in the MMP assay buffer. Subsequently, the fluorescence intensity of Rh123 was monitored using a Shimadzu RF-5000U fluorescence spectrophotometer at the excitation and emission wavelength of 490 nm and 535 nm, respectively (43).

*Determination of mitochondrial swelling*

Changes in light scattering were used to analyze mitochondrial swelling. For this purpose, samples containing isolated mitochondria (0.5 mg protein/ml) were mixed with a swelling buffer comprising 70 mM sucrose, 230 mM mannitol, 3 mM HEPES, 2 mM trisphosphate, 5 mM succinate, and 1 μM of rotenone and incubated at 30 ^°^C. The absorbance was measured at 549 nm at 10-minute intervals with an ELISA reader (Tecan, Rainbow Thermo, Austria). A decrease in absorbance indicates an increase in mitochondrial swelling (43).

### Sperm characteristics assay

*Sperm motility*

To collect viable epididymal sperms, the epididymis was minced in 5 ml of Ham’s F10 solution and then incubated for five minutes at 37 ^°^C in 5% CO_2_. One drop of the sperm suspension was placed on a glass slide. The sperm count was evaluated twice for each sample using a microscope at ×40 magnification, and the means were calculated. At least 200 sperm were examined per animal. The percentage of motile sperm is then calculated as a proportion of total sperm in each replicate (44).

*Sperm count*

The two epididymis were finely chopped in distilled water to collect epididymal spermatozoa. The resulting diluted sperm suspension was carefully transferred to a physiological saline solution and incubated at 32 ^°^C for ten minutes. Five milliliters of the diluted sperm suspension were placed onto each Neubauer hemocytometer slide. The Spermatozoa were quantified using light microscopy at a magnification of ×40 in five specified squares. The resulting numbers were expressed as 1 million in a 1 ml sample (44).

*Sperm morphological abnormalities*

To investigate morphological abnormalities, sperm smears were prepared on clean slides without grease residue. The prepared slides were then left to air dry throughout the night. Subsequently, the slides were stained with 1% eosin-Y/5% nigrosin. The specimens were carefully examined under a microscope at ×100 magnification for morphological abnormalities, including amorphous shapes, bicephalic heads, hookless structures, coiled formations, and irregular tails. At least 200 sperm per animal were analyzed, and the percentage of abnormal sperm was calculated (44).

### Histopathological examination

Testis tissue samples underwent fixation in 10% formalin, dehydration, and embedding in paraffin. These embedded samples were then cut into thin sections, deparaffinized, and stained with hematoxylin and eosin for examination under a light microscope (45).

### Statistical analysis

Data from all experiments were independently replicated three times, and the results are presented as mean± standard deviation (SD). Data was analyzed and plotted using GraphPad Prism 9.0 software. One-way ANOVA, followed by the Tukey multiple comparison test, was used to determine the significance between groups. *P*-values<0.05 were considered statistically significant.

## Results

### Effects of CUR on VPA-induced lipid peroxidation in mice testis

**Figure 1:**
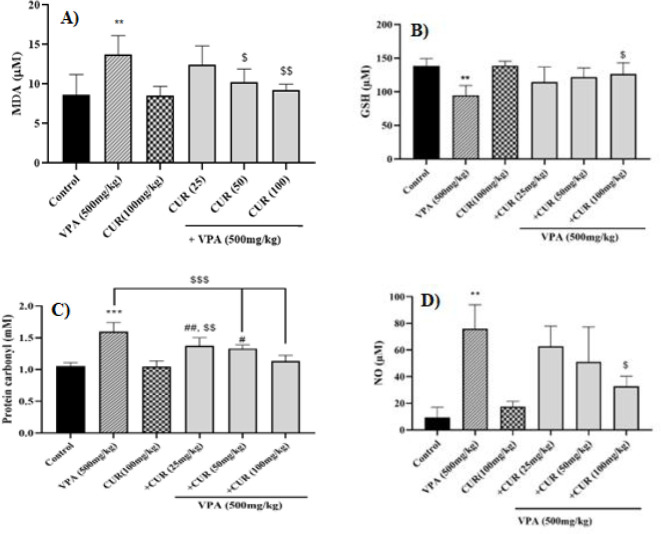
Protective effects of curcumin on valproate-induced oxidative stress

Increasing MDA level is considered a significant indicator of oxidative damage to the cell membrane. As shown in Figure 1A, the MDA level was raised (*P*<0.01) in mice receiving VPA compared to the control group in testis tissue. The results indicated that in the groups administered with 50 and 100 mg/kg of CUR, the level of MDA was notably lower than that of the group receiving VPA. Also, no significant difference was seen between the doses of CUR (Figure 1A).

### Effects of VPA and CUR on GSH concentration in mice testis tissue

Oxidative stress-induced organ damage is often attributed to the disparity between ROS levels and antioxidants, such as GSH. The result revealed a noticeable reduction in the levels of GSH in the testicular tissue of mice treated with VPA, as compared to the control group. Furthermore, a statistically significant elevation (*P*<0.05) in GSH concentration was observed in the testis tissues of animals administered with 100 mg/kg of CUR compared to the group subjected to VPA. There was no significant difference between different doses of CUR (Figure 1B). 

### Effects of CUR on VPA-induced protein carbonyl in mice testis

Compared to the control group, the group receiving VPA demonstrated a significantly higher protein carbonyl content (*P*<0.001). As depicted in Figure 3, all CUR doses significantly reduced protein carbonyl content compared to the group that received VPA in mice testicular tissue. Also, there was a significant difference between the dose of 25 mg/kg (*P*<0.05) and 50 mg/kg (*P*<0.01) compared to the dose of 100 mg/kg of CUR (Figure 1C).

### Effects of VPA and CUR on NO level in mice testis

As illustrated in Figure 4, the administration of VPA resulted in a notable elevation in tissue NO levels compared to the control group. Conversely, animals receiving 100 mg/kg CUR exhibited a lower NO content than the VPA group (Figure 1D). No significant difference was seen between different doses of CUR (Figure 1D).

### Effects of VPA and CUR on TNF-ɑ and IL-6 levels in mice testis tissue

**Figure 2:**
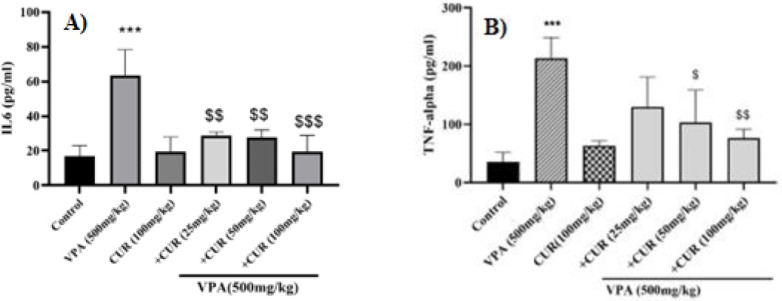
Protective effects of curcumin on valproate-induced inflammation

Based on the findings presented in Figure 2, mice treated with VPA displayed a significant rise in inflammatory mediators in testis tissue. As evident in Figure 2A, IL6 concentration significantly increased compared to the control group (*P*<0.001). CUR at all used doses significantly inhibited VPA-induced increase in IL-6 level, but there was no significant difference between the three doses (Figure 2A).

TNF-ɑ levels were compared to the control group. This elevation of TNF-ɑ is indicative of inflammation in the testis. Furthermore, there was a substantial reduction in TNF-ɑ concentration in the animals that received 50 and 100 mg/kg of CUR compared to the VPA group. Comparison of the effects of three doses of TNF-ɑ showed a significant (*P*<0.01) difference between the dose of 25 and 100 mg/kg (Figure 2B).

### Effects of CUR on VPA-induced mitochondrial dysfunction in mice testis tissue

**Figure 3:**
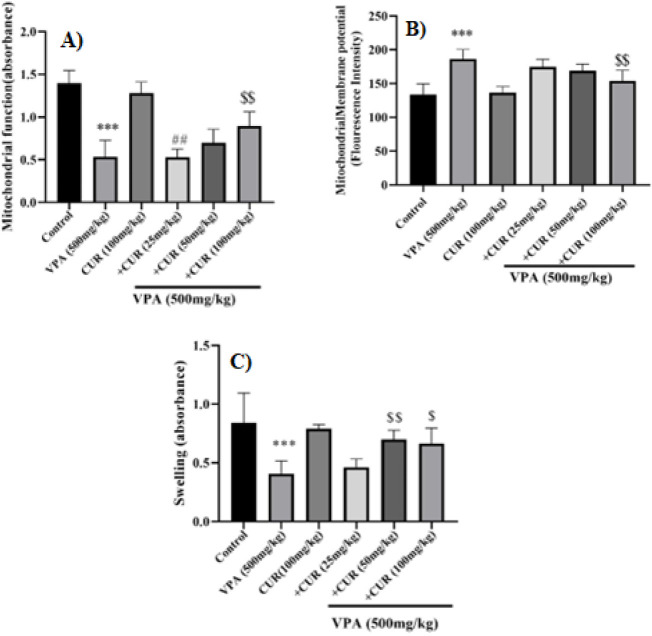
Protective effects of curcumin on valproate-induced mitochondrial toxicity

Mitochondrial function was assessed using an MTT assay based on the ability of healthy mitochondria to convert MTT to MTT formazon. According to the results, mitochondrial function was disrupted in mice testicular tissues that received VPA compared to the control group (*P*<0.001). On the other hand, CUR at a dose of 100 mg/kg effectively mitigated the harmful effects of valproate. Figure 3A shows a significant difference between the dose of 25 and 100 mg/kg of CUR (*P*<0.01).

### Effects of VPA and CUR on mitochondrial membrane potential in mice testis tissue

Mitochondrial membrane potential (MMP) was assayed by rhodamine 123, which decrease in fluorescence intensity indicates MMP collapse and serves as an initial marker of mitochondrial dysfunction. VPA considerably reduced the MMP compared to the control group, as illustrated in Figure 3B (*P*<0.001). Conversely, the addition of CUR (100 mg/kg) inhibited VPA-induced MMP collapse when compared to the VPA group (*P*<0.01) ( Figure 3B).

### Effects of CUR on VPA-induced mitochondrial swelling in mice testis tissue

Mitochondrial swelling is considered the leading indicator of mitochondrial membrane permeability. An increase in mitochondrial swelling is associated with a decrease in absorbance. As depicted in Figure 3C, VPA caused an elevation in the level of mitochondrial inflammation compared to the control group (*P*<0.001). On the other hand, administration of CUR significantly alleviated mitochondrial swelling compared to the VPA group (Figure 3C).

### Effects of VPA and CUR on the sperm characteristics

*Sperm count*

Epididymal sperm analysis in the VPA-treated group indicated a considerable decline in sperm count compared to the control group (*P*<0.001). Interestingly, the group treated with high doses of CUR (100 mg/kg) had a considerable increase in sperm count as compared to the VPA group (*P*<0.01)(Table 1).

*Sperm motility*

The group that received VPA exhibited a notable decrease in sperm motility (*P*<0.001). However, there was a slight improvement in sperm motility in the group that received a high dose of CUR (100 mg/kg) when compared to the VPA-treated group (*P*<0.050)(Table 1).

*Sperm morphological abnormalities*

VPA remarkably raised the percentage of sperm abnormality compared to the control group (*P*<0.001). Conversely, cotreatment with a high dose of CUR (100 mg/kg) effectively reduced the occurrence of abnormal sperm in comparison to the group receiving VPA (*P*<0.001)( Table 1).

### Histopathologic manifestation

**Figure 4:**
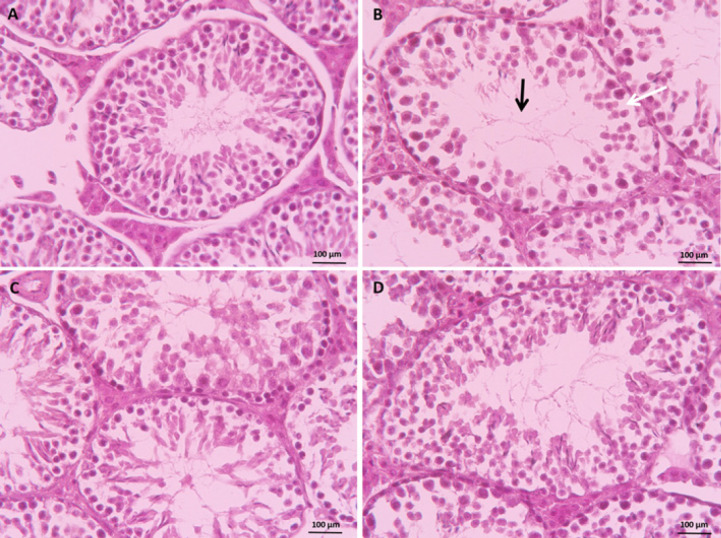
Protective effects of curcumin on valproate-induced testis injury

As illustrated in Figure 4-A, the interstitial and Sertoli cells in the control group remained normal, and the tubules exhibited their typical structure without any signs of disruption. On the other hand, analysis of testicular tissue in the VPA-treated group revealed severe histopathological damages, including an enlarged diameter of the seminiferous tubule lumen, detachment of the spermatogenic cell lineage from the basal membrane, and a decrease in the thickness of the epithelium of the seminiferous tubule (Figure 4-B). However, CUR significantly improved testicular features at 100 and 50 mg/kg (Figures 4-C and D) compared to the VPA-treated group.

## Discussion

The current study showed that CUR could ameliorate VPA testis toxicity by inhibiting oxidative stress and inflammation pathways. This study showed that CUR improved mitochondrial function, antioxidant level, and attenuation of the level of inflammatory markers and oxidative damage in mice subjected to VPA. Also, sperm and histopathological changes were observed in the animals that received VPA, and treatment with CUR ameliorated these changes. 

VPA is a frequently used medication for the treatment of epilepsy and bipolar disorder. Extensive studies have shown that prolonged use of VPA is associated with detrimental effects on the reproductive system (46).

Several research investigations have demonstrated the potential of various antioxidants, including lycopene. (47), flavonoids, vitamin E, and vitamin C (48) to mitigate the harmful effects associated with VPA. A study conducted by Mehmood *et al*. revealed that the extract of Momordica cochinchinensis, possessing antioxidant properties, effectively averts the detrimental effects of VPA on testicular tissue (49).

CUR is obtained from the rhizome of *C. longa* (50) and is a non-enzymatic antioxidant characterized by its phenolic groups (51). It exhibits numerous beneficial properties, such as antioxidant and anti-inflammatory activities, and is utilized in treating various inflammatory conditions. (52). Additionally, studies have shown that CUR exerts a significant protective effect in diminishing the testicular toxicity caused by aflatoxin (53) , cadmium (33), and Lindane (54). So, this study aimed to explore the potential protective effects of CUR in preventing reproductive toxicity induced by VPA in male mice.

The exact mechanism by which valproic acid (VPA) impairs reproductive function remains unclear, but research indicates that oxidative stress and mitochondrial dysfunction are key factors in its reproductive toxicity.

VPA has been shown to inhibit mitochondrial respiration, reducing ATP production and increasing reactive oxygen species (ROS), lipid peroxidation, and glutathione (GSH) oxidation. CMX *et al*. found that VPA treatment in mice led to oxidative damage to proteins and lipids, decreased serum testosterone levels, and GSH depletion in testicular tissue (55).

Our research also observed increased lipid peroxidation, protein carbonyl levels, and decreased GSH in VPA-treated mice, indicating oxidative damage. CUR exacerbated these alterations, but CUR treatment helped reduce malondialdehyde (MDA) levels and improved GSH concentration. CUR’s antioxidant properties enhance GSH synthesis and mitigate oxidative stress compared to VPA alone. The study also showed a reduction in protein carbonyl levels, confirming CUR’s protective effects.

Various studies have indicated that inflammation contributes to tissue toxicity caused by VPA (55). On the other hand, oxidative stress and inflammation are intimately linked processes and often exhibit a bidirectional relationship. Oxidative stress can stimulate pro-inflammatory signaling pathways, resulting in the release of inflammatory mediators. Conversely, inflammation can also trigger oxidative stress by activating immune cells, producing ROS and RNS (56). 

VPA treatment significantly elevated TNF-α levels, a marker of inflammation, which is consistent with previous studies linking inflammation to VPA-induced toxicity (57). CUR administration reduced TNF-α levels, aligning with prior research showing that CUR can inhibit pro-inflammatory pathways and reduce the expression of TNF-α in various tissue models (58). The anti-inflammatory effects of CUR are likely mediated through its modulation of NF-κB signaling and its ability to suppress the release of inflammatory cytokines. For example, CUR has been demonstrated to lower TNF-α levels in groups exposed to nicotine (58) . Previous studies have indicated that excessive NO production amplifies ROS’s impact, leading to increased oxidative stress (59). Additionally, other investigations have shown that VPA notably elevates NO levels. At the same time, researchers highlighted the ability of CUR to diminish NO levels (60). Our findings align with these findings, demonstrating that CUR reduces NO levels.

Mitochondria are key players in cellular energy production and ROS regulation. VPA-induced mitochondrial dysfunction, including mitochondrial swelling and a collapse in MMP, is a hallmark of testicular toxicity. CUR treatment significantly improved mitochondrial function and reduced swelling, which may be attributed to its antioxidant properties that prevent oxidative damage to mitochondrial membranes and preserve MMP integrity. Previous studies have similarly demonstrated CUR’s protective effects on mitochondrial function in cardiac and renal tissues (61).

Therefore, any impairment to the integrity of mitochondria can adversely affect testicular function, particularly in sperm production. A structural alteration would happen following the VPA-induced oxidation of the thiol group at the mitochondrial permeability transition (MPT) pores(62). MPT pore opening leads to MMP loss. Our investigation found increased testicular mitochondrial dysfunction and swelling in VPA-treated groups, aligning with previous studies. In contrast, CUR treatment improved mitochondrial function and swelling. Past research also shows CUR’s protective effects on cardiac and renal mitochondria, supporting our findings.

Ganjkhani *et al*. reported that administering VPA for 30 consecutive days resulted in a significant decrease in testicular weight and sperm count and a reduction in normal sperm morphology in rats (63). Previous studies have also indicated that 40 to 80 percent of infertile men exhibit elevated ROS levels. Indeed, there is a direct correlation between oxidative stress and sperm damage (64) Elevated ROS levels and the oxidation of testicular membrane lipids can adversely affect various aspects of sperm quality, such as count, morphology, motility, and survival (65) Moreover, the reduction of glutathione level caused by ROS leads to disruption in the integrity of sperm membranes, consequently impairing sperm function (66) In the current study, we observed that VPA treatment resulted in reproductive toxicity, characterized by decreased sperm count, motility, and normal morphology. This is while the administration of CUR led to a reduction in valproate toxicity on sperm parameters, which is consistent with previous studies. Zowail *et al*. found that CUR in cadmium-treated mice prevented spermatogenic injury and reduced sperm count (67) Another study by Rezvanfar *et al*. revealed that simultaneous intake of CUR and nicotine is associated with a notable decrease in ROS, lipid peroxidation, and TNF-α, as well as improvement in sperm motility, count, and morphology (68)

We observed that VPA caused severe testicular tissue damage, which was mitigated by the administration of CUR. This included an increase in the diameter of seminiferous tubules, inhibition of the detachment of spermatogenic cell layers from the basal membrane, and a reduction in the thickness of the seminiferous epithelium.

In summary, this study demonstrated the attenuation effect of CUR on testis toxicity induced by VPA through amelioration of oxidative stress and inflammatory conditions. Nevertheless, the current study had some limitations. First, the involvement of various mechanisms, such as mitochondrial toxicity, oxidative stress, and inflammation, was insufficiently elaborated by the current data, and the assessment of other factors, such as NF-κB signaling, could be helpful. Second, testis toxicity can be evaluated in more detail using the Johnson score. Despite these limitations, to our knowledge, this is still the first study showing the protective effect of CUR against VPA, and the underlying mechanism, including inhibiting oxidative stress, mitochondrial toxicity, and inflammation, was evaluated.

Oxidative stress assayed in groups including control, valproate (VPA 500 mg/kg), curcumin alone (CUR 100 mg/kg), valproate with different doses of curcumin (CUR 25, 50, and 100 mg/kg) in male mice (n=6/group) for 8 weeks via IP injection. Oxidative stress markers, including A) lipid peroxidation, B) Glutathione content, C) protein carbonyl concentration, and D) Nitric Oxide concentration, were assayed in mice’s testicular tissue. Data are expressed as the mean±SD. ^∗∗^*P<*0.01 and ^∗∗^∗*P<*0.001, significantly different in comparison with the control group. ^$^*P<*0.05, ^$$^*P<*0.01, ^$$$^*P<*0.001, in comparison with the VPA group. ^#^*P<*0.05, ^##^*P<*0.01, in comparison with the CUR (100)+VPA group

VPA (valproate), CUR (Curcumin), Glutathione (GSH), Malondialdehyde (MDA), nitric oxide (NO)

Inflammation assayed in groups including control, valproate (VPA 500 mg/kg), curcumin alone (CUR 100 mg/kg), valproate with different doses of curcumin (CUR 25, 50, and 100 mg/kg) in male mice (n=6/group) for 8 weeks via IP injection. Inflammatory markers, including A) IL-6 and B) TNF-alpha, were assayed in mice’s testicular tissue. Data are expressed as the mean±SD. ^∗∗∗^*P<*0.001, relative to the control group. ^$^*P<*0.05, ^$$^*P<*0.01, ^$$$^*P<*0.001 relative to the VPA group

VPA (valproate), CUR (Curcumin)

Mitochondrial toxicity assayed in groups including control, valproate (VPA 500 mg/kg), curcumin alone (CUR 100 mg/kg), valproate with different doses of curcumin (CUR 25, 50, and 100 mg/kg) in male mice (n=6 group) for 8 weeks via IP injection. Mitochondrial toxicity markers, including A) mitochondrial function, B) mitochondrial membrane potential, and C) mitochondrial swelling, were assayed in mice’s testicular tissue. Data are expressed as the mean±SD. ^∗∗^*P<*0.01 and ^∗∗∗^*P<*0.001, relative to the control group. ^$^*P<*0.05, ^$$^*P<*0.01 relative to the VPA group. ^##^*P<*0.01, compared with the CUR (100)+VPA group

VPA (valproate), CUR (Curcumin)

Testis injury assayed in groups including control, valproate (VPA 500 mg/kg), curcumin alone (CUR 100 mg/kg), valproate with different doses of curcumin (CUR 25, 50, and 100 mg/kg) in male mice (n=6/group) for 8 weeks via IP injection. Hematoxylin- and eosin-stained section of mice testis: the ameliorative effects of different doses of curcumin against valproate-induced histopathological damages in the testicular tissue (400×); A) control group revealed normal features and Intertubular cells and Sertoli cells were normal, and the tubules were not disrupted. B) In the sodium valproate-treated group (panel B), histopathological abnormalities such as an increase in the diameter of the seminiferous tubule lumen (black arrow), separation of cells in the spermatogenic lineage from the basal membrane, and a decrease in the thickness of the epithelium of the seminiferous tubule (white arrow) were observed. In contrast, the group treated with curcumin at doses of 100 and 50 mg/kg (panel C and D) showed a significant improvement in testicular features compared to the sodium valproate-treated group

**Table 1:** Effects of curcumin on valproate-induced changes in mice’s sperm parameters

	**Control**	**VPA** **(500 mg/kg)**	**CUR** **(100 mg/kg)**	**VPA+CUR ** **(25 mg/kg)**	**VPA+CUR ** **(50 mg/kg)**	**VPA+CUR ** **(100 mg/kg)**
**Count (10^6 milion/ml)**	28.33±2.1	16.67±1.7***	28.83±3.3	18.67±5.2	20.83±2.8	24.17±4^$$^
**Motility (%)**	68.17±9	27.5±13.6***	60.17±910.5	30.17±16	35±97.3	45.67±8.2
**Sperm abnormality (%)**	7.16±3.3	23±3.6***	8.83±3.9	17.1±3.3^$^	18±1.8	10.83±1.4^$$$^

Treatments were applied for 8 weeks via IP injection. Sperm analysis was done in groups including control, valproate (VPA 500 mg/kg), curcumin alone (CUR 100 mg/kg), valproate with different doses of curcumin (CUR 25, 50, and 100 mg/kg) in male mice (n=6/group).Values are expressed as mean±SD; ***significantly different compared to the control group (*P<*0.001), $ significantly different compared to the valproate group (*P<*0.05), $$ significantly different compared to the valproate group (*P<*0.01), $$$ significantly different compared to the valproate group (*P<*0.001)

CUR: curcumin, VPA: valproate

## Conclusion

According to our results, VPA induced oxidative stress and elevated inflammation in the testicular tissue of mice. In addition, it was noted that VPA has been associated with a reduction in sperm motility and count, along with an increase in the occurrence of abnormal sperm. The data of this study indicated that the administration of CUR leads to a reduction in reproductive toxicity induced by VPA. CUR can provide substantial protection without any adverse effects. It can potentially alleviate oxidative stress-mediated damage to testicular mitochondria and tissue in mice by inhibiting intracellular ROS production. Hence, it is recommended that CUR be considered a supplement for patients undergoing long-term treatment with VPA. However, additional clinical research is needed to substantiate the protective role of CUR on mitochondrial function.

## Declaration

We have not used any AI tools or technologies to prepare this manuscript.
